# Research and Practice of AI Ethics: A Case Study Approach Juxtaposing Academic Discourse with Organisational Reality

**DOI:** 10.1007/s11948-021-00293-x

**Published:** 2021-03-08

**Authors:** Mark Ryan, Josephina Antoniou, Laurence Brooks, Tilimbe Jiya, Kevin Macnish, Bernd Stahl

**Affiliations:** 1 Wageningen Economic Research, Wageningen University and Research, Wageningen, The Netherlands; 2 UCLan Cyprus, Larnaka, Cyprus; 3 De Montford University, Leicester, UK; 4 Northampton University, Northampton, UK; 5 The University of Twente, Enschede, The Netherlands

**Keywords:** Smart information systems, Big data analytics, Artificial intelligence ethics, Multiple-case study analysis, Philosophy of technology

## Abstract

This study investigates the ethical use of Big Data and Artificial Intelligence (AI) technologies (BD + AI)—using an empirical approach. The paper categorises the current literature and presents a multi-case study of 'on-the-ground' ethical issues that uses qualitative tools to analyse findings from ten targeted case-studies from a range of domains. The analysis coalesces identified singular ethical issues, (from the literature), into clusters to offer a comparison with the proposed classification in the literature. The results show that despite the variety of different social domains, fields, and applications of AI, there is overlap and correlation between the organisations’ ethical concerns. This more detailed understanding of ethics in AI + BD is required to ensure that the multitude of suggested ways of addressing them can be targeted and succeed in mitigating the pertinent ethical issues that are often discussed in the literature.

## Introduction

Big Data and Artificial Intelligence (BD + AI) are emerging technologies that offer great potential for business, healthcare, the public sector, and development agencies alike. The increasing impact of these two technologies and their combined potential in these sectors can be highlighted for diverse organisational aspects such as for customisation of organisational processes and for automated decision making. The combination of Big Data and AI, often in the form of machine learning applications, can better exploit the granularity of data and analyse it to offer better insights into behaviours, incidents, and risk, eventually aiming at positive organisational transformation.

Big Data offers fresh and interesting insights into structural patterns, anomalies, and decision-making in a broad range of different applications (Cuquet & Fensel, 2018), while AI provides predictive foresight, intelligent recommendations, and sophisticated modelling. The integration and combination of AI + BD offer phenomenal potential for correlating, predicting and prescribing recommendations in insurance, human resources (HR), agriculture, and energy, as well as many other sectors. While BD + AI provides a wide range of benefits, they also pose risks to users, including but not limited to privacy infringements, threats of unemployment, discrimination, security concerns, and increasing inequalities (O’Neil, 2016).^1^ Adequate and timely policy needs to be implemented to prevent many of these risks occurring.

One of the main limitations preventing key decision-making for ethical BD + AI use is that there are few rigorous empirical studies carried out on the ethical implications of these technologies across multiple application domains. This renders it difficult for policymakers and developers to identify when ethical issues resulting from BD + AI use are only relevant for isolated domains and applications, or whether there are repeated/universal concerns which can be seen across different sectors. While the field lacks literature evaluating ethical issues^2^ ‘on the ground’, there are even fewer multi-case evaluations.

This paper provides a cohesive multi-case study analysis across ten different application domains, including domains such as government, agriculture, insurance, and the media. It reviews ethical concerns found within these case studies to establish cross-cutting thematic issues arising from the implementation and use of BD + AI. The paper collects relevant literature and proposes a simple classification of ethical issues (short term, medium term, long term), which is then juxtaposed with the ethical concerns highlighted from the multiple-case study analysis. This multiple-case study analysis of BD + AI offers an understanding of current organisational practices.

The work described in this paper makes an important contribution to the literature, based on its empirical findings. By presenting the ethical issues across an array of application areas, the paper provides much-needed rigorous empirical insight into the social and organisational reality of ethics of AI + BD. Our empirical research brings together a collection of domains that gives a broad oversight about issues that underpin the implementation of AI. Through its empirical insights the paper provides a basis for a broader discussion of how these issues can and should be addressed.

This paper is structured in six main sections: this introduction is followed by a literature review, which allows for an integrated review of ethical issues, contrasting them with those found in the cases. This provides the basis for a categorisation or classification of ethical issues in BD + AI. The third section contains a description of the interpretivist qualitative case study methodology used in this paper. The subsequent section provides an overview of the organisations participating in the cases to contrast similarities and divisions, while also comparing the diversity of their use of BD + AI.^3^ The fifth section provides a detailed analysis of the ethical issues derived from using BD + AI, as identified in the cases. The concluding section analyses the differences between theoretical and empirical work and spells out implications and further work.

## Literature Review

An initial challenge that any researcher faces when investigating ethical issues of AI + BD is that, due to the popularity of the topic, there is a vast and rapidly growing literature to be considered. Ethical issues of AI + BD are covered by a number of academic venues, including some specific ones such as the AAAI/ACM Conference on AI, Ethics, and Society (https://dl.acm.org/doi/proceedings/10.1145/3306618), policy initiative and many publicly and privately financed research reports (Whittlestone, Nyrup, Alexandrova, Dihal, & Cave, 2019). Initial attempts to provide overviews of the area have been published (Jobin, 2019; Mittelstadt, Allo, Taddeo, Wachter, & Floridi, 2016), but there is no settled view on what counts as an ethical issue and why. In this paper we aim to provide a broad overview of issues found through the case studies. This paper puts forward what are commonly perceived to be ethical issues within the literature or concerns that have ethical impacts and repercussions. We explicitly do not apply a particular philosophical framework of ethics but accept as ethical issues those issues that we encounter in the literature. This review is based on an understanding of the current state of the literature by the paper's authors. It is not a structured review and does not claim comprehensive coverage but does share some interesting insights.

**Fig. 1:**
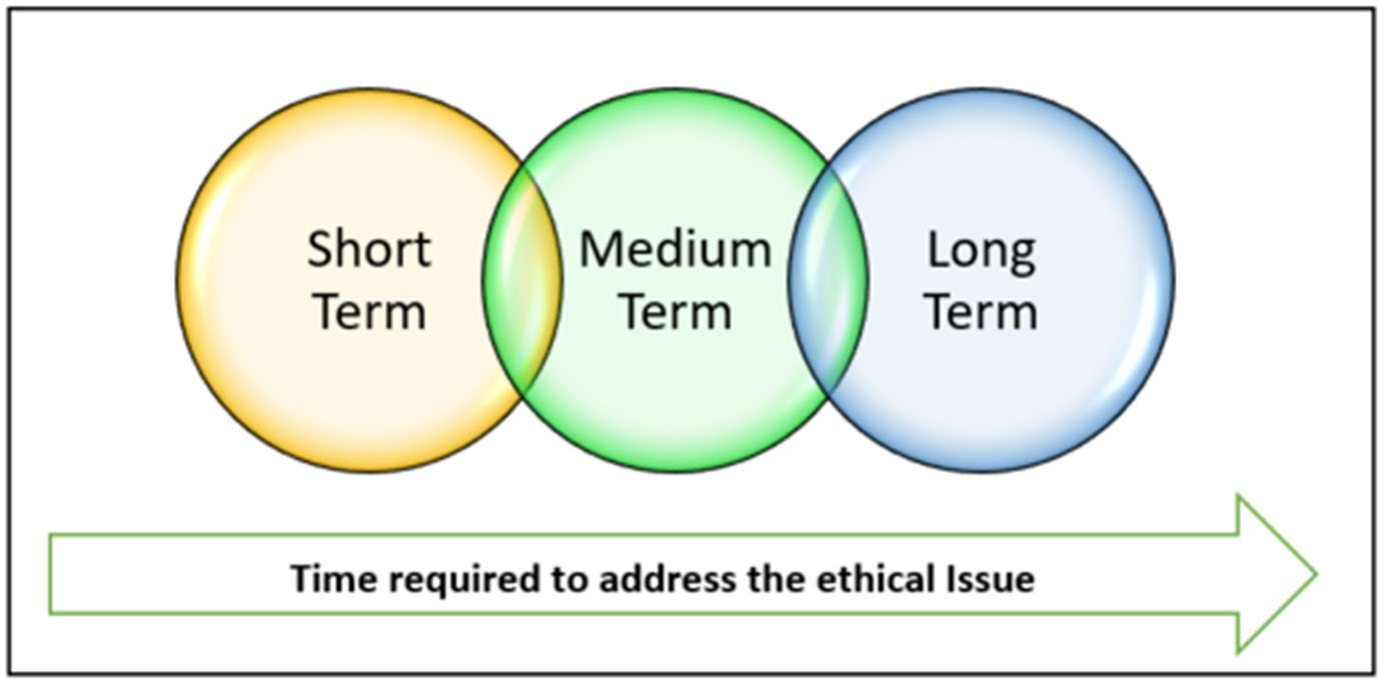
Temporal horizon for addressing ethical issues

To be able to undertake the analysis of ethical issues in our case studies, we sought to categorise the ethical issues found in the literature. There are potentially numerous ways of doing so and our suggestion does not claim to be authoritative. Our suggestion is to order ethical issues in terms of their temporal horizon, i.e., the amount of time it is likely to take to be able to address them. Time is a continuous variable, but we suggest that it is possible to sort the issues into three clusters: short term, medium term, and long term (see Fig. 1).

*As* suggested by Baum (2017), it is best to acknowledge that there will be ethical issues and related mitigating activities that cannot exclusively fit in as short, medium or long term.

ather than seeing it as an authoritative classification, we see this as a heuristic that reflects aspects of the current discussion. One reason why this categorisation is useful is that the temporal horizon of ethical issues is a potentially useful variable, with companies often being accused of favouring short-term gains over long-term benefits. Similarly, short-term issues must be able to be addressed on the local level for short-term fixes to work.

### Short-term issues

These are issues for which there is a reasonable assumption that they are capable of being addressed in the short term. We do not wish to quantify what exactly counts as short term, as any definition put forward will be contentious when analysing the boundaries and transition periods. A better definition of short term might therefore be that such issues can be expected to be successfully addressed in technical systems that are currently in operation or development. Many of the issues we discuss under the heading of short-term issues are directly linked to some of the key technologies driving the current AI debate, notably machine learning and some of its enabling techniques and approaches such as neural networks and reinforcement learning.

Many of the advantages promised by BD + AI involve the use of personal data, data which can be used to identify individuals. This includes health data; customer data; ANPR data (Automated Number Plate Recognition); bank data; and even includes data about farmers’ land, livestock, and harvests. Issues surrounding *privacy* and *control of data* are widely discussed and recognized as major ethical concerns that need to be addressed (Boyd & Crawford, 2012; Tene & Polonetsky, 2012, 2013; Mittelstadt, Allo, Taddeo, Wachter, & Floridi, 2016; Jain, Gyanchandani, & Khare, 2016; Mai, 2016; Macnish, 2018). The concern surrounding privacy can be put down to a combination of a general level of awareness of privacy issues and the recently-introduced General Data Protection Regulation (GDPR). Closely aligned with privacy issues are those relating to *transparency* of processes dealing with data, which can often be classified as *internal, external, and deliberate opaqueness* (Burrell, 2016; Lepri, Staiano, Sangokoya, Letouzé, & Oliver, 2017; Mittelstadt, Allo, Taddeo, Wachter, & Floridi, 2016).

The *Guidelines for Trustworthy AI*^4^ were released in 2018 by the High-Level Expert Group on Artificial Intelligence (AI HLEG^5^), and address the need for technical robustness and safety, including accuracy, reproducibility, and reliability. *Reliability* is further linked to the requirements of *diversity, fairness, and social impact* because it addresses freedom from bias from a technical point of view. The concept of reliability, when it comes to BD + AI, refers to the capability to verify the stability or consistency of a set of results (Bush, 2012; Ferraggine, Doorn, & Rivera, 2009; Meeker and Hong, 2014).

If a technology is unreliable, error-prone, and unfit-for-purpose, adverse ethical issues may result from decisions made by the technology. The accuracy of recommendations made by BD + AI is a direct consequence of the degree of reliability of the technology (Barolli, Takizawa, Xhafa, & Enokido, 2019). *Bias and discrimination* in algorithms may be introduced consciously or unconsciously by those employing the BD + AI or because of algorithms reflecting pre-existing biases (Baroccas and Selbst, 2016). Examples of bias have been documented often reflecting “an imbalance in socio-economic or other ‘class’ categories—ie, a certain group or groups are not sampled as much as others or at all” (Panch et al., 2019). have the potential to affect levels of inequality and discrimination, and if biases are not corrected these systems can reproduce existing patterns of discrimination and inherit the prejudices of prior decision makers (Barocas & Selbst, 2016, p. 674). An example of inherited prejudices is documented in the United States, where African-American citizens, more often than not, have been given longer prison sentences than Caucasians for the same crime.

### Medium-term issues

Medium-term issues are not clearly linked to a particular technology but typically arise from the integration of AI techniques including machine learning into larger socio-technical systems and contexts. They are thus related to the way life in modern societies is affected by new technologies. These can be based on the specific issues listed above but have their main impact on the societal level. The use of BD + AI may allow individuals’ behaviour to be put under scrutiny and *surveillance*, leading to infringements on *privacy, freedom, autonomy, and self-determination* (Wolf, 2015). There is also the possibility that the increased use of algorithmic methods for societal decision-making may create a type of *technocratic governance* (Couldry & Powell, 2014; Janssen & Kuk, 2016), which could infringe on people’s decision-making processes (Kuriakose & Iyer, 2018). For example, because of the high levels of public data retrieval, BD + AI may harm people’s *freedom of expression, association, and movement, through fear of surveillance and chilling effects* (Latonero, 2018).

Corporations have a responsibility to the end-user to ensure *compliance, accountability, and transparency* of their BD + AI (Mittelstadt, Allo, Taddeo, Wachter, & Floridi, 2016). However, when the source of a problem is difficult to trace, owing to issues of opacity, it becomes challenging to identify who is responsible for the decisions made by the BD + AI. It is worth noting that a large-scale survey in Australia in 2020 indicated that 57.9% of end-users are not at all confident that most companies take adequate steps to protect user data. The significance of understanding and employing responsibility is an issue targeted in many studies (Chatfield et al., 2017; Fothergill et al., 2019; Jirotka et al., 2017; Pellé & Reber, 2015). Trust and control over BD + AI as an issue is reiterated by a recent ICO report demonstrating that most UK citizens do not trust organisations with their data (ICO, 2017).

*Justice* is a central concern in BD + AI (Johnson, 2014, 2018). As a starting point, justice consists in giving each person his or her due or treating people equitably (De George, p. 101). A key concern is that benefits will be reaped by powerful individuals and organisations, while the burden falls predominantly on poorer members of society (Taylor, 2017). BD + AI can also reflect human intentionality, deploying patterns of *power and authority* (Portmess & Tower, 2015, p. 1). The knowledge offered by BD + AI is often in the hands of a few powerful corporations (Wheeler, 2016). Power imbalances are heightened because companies and governments can deploy BD + AI for surveillance, privacy invasions and manipulation, through personalised marketing efforts and social control strategies (Lepri, Staiano, Sangokoya, Letouzé, & Oliver, 2017, p. 11). They play a role in the ascent of datafication, especially when specific groups (such as corporate, academic, and state institutions) have greater unrestrained access to big datasets (van Dijck, 2014, p. 203).

*Discrimination*, in BD + AI use, can occur when individuals are profiled based on their online choices and behaviour, but also their gender, ethnicity and belonging to specific groups (Calders, Kamiran, & Pechenizkiy, 2009; Cohen et al., 2014; and Danna & Gandy, 2002). Data-driven algorithmic decision-making may lead to discrimination that is then adopted by decision-makers and those in power (Lepri, Staiano, Sangokoya, Letouzé, & Oliver, 2017, p. 4). Biases and discrimination can contribute to *inequality*. Some groups that are already disadvantaged may face worse inequalities, especially if those belonging to historically marginalised groups have less *access and representation* (Barocas & Selbst, 2016, p. 685; Schradie, 2017). Inequality-enhancing biases can be reproduced in BD + AI, such as the use of predictive policing to target neighbourhoods of largely ethnic minorities or historically marginalised groups (O’Neil, 2016).

BD + AI offers great potential for increasing profit, reducing physical burdens on staff, and employing innovative sustainability practices (Badri, Boudreau-Trudel, & Souissi, 2018). They offer the potential to bring about improvements in innovation, science, and knowledge; allowing organisations to progress, expand, and economically benefit from their development and application (Crawford et al., 2014). BD + AI are being heralded as monumental for the economic growth and development of a wide diversity of industries around the world (Einav & Levin, 2014). The economic benefits accrued from BD + AI may be the strongest driver for their use, but BD + AI also holds the potential to cause *economic harm* to citizens and businesses or create other adverse ethical issues (Newman, 2013).

However, some in the literature view the co-development of employment and automation as somewhat naïve outlook (Zuboff, 2015). BD + AI companies may benefit from a ‘post-labour’ automation economy, which may have a negative impact on the labour market (Bossman, 2016), replacing up to 47% of all US jobs within the next 20 years (Frey & Osborne, 2017). The professions most at risk of *affecting employment* correlated with three of our case studies: farming, administration support and the insurance sector (Frey & Osborne, 2017).

### Long-term issues

Long-term issues are those pertaining to fundamental aspects of nature of reality, society, or humanity. For example, that AI will develop capabilities far exceeding human beings (Kurzweil, 2006). At this point, sometimes called the ‘*singularity*’ machines achieve human intelligence, are expected to be able to improve on themselves and thereby surpass human intelligence and become *superintelligent* (Bostrom, 2016). If this were to happen, then it might have dystopian consequences for humanity as often depicted in science fiction. Also, it stands to reason that the superintelligent, or even just the normally intelligent machines may acquire a moral status.

It should be clear that these expectations are not universally shared. They refer to what is often called ‘ artificial general intelligence’ (AGI), a set of technologies that emulate human reasoning capacities more broadly.^6^

Furthermore, if we may acquire new capabilities, e.g. by using technical implants to enhance human nature. The resulting being might be called a *transhuman*, the next step of human evolution or development. Again, it is important to underline that this is a contested idea (Livingstone, 2015) but one that has increasing traction in public discourse and popular science accounts (Harari, 2017).

We chose this distinction of three groups of issues for understanding how mitigation strategies within organisations can be contextualised. We concede that this is one reading of the literature and that many others are possible. In this account of the literature we tried to make sense of the current discourse to allow us to understand our empirical findings which are introduced in the following sections.

## Case Study Methodology

Despite the impressive amount of research undertaken on ethical issues of AI + BD (e.g. Mittelstadt, Allo, Taddeo, Wachter, & Floridi, 2016; Zwitter, 2014), there are few case studies exploring such issues. This paper builds upon this research and employs an interpretivist methodology to do so, focusing on how, what, and why questions relevant to the ethical use of BD + AI (Walsham, 1995a, b). The primary research questions for the case studies were: How do organisations perceive ethical concerns related to BD + AI and in what ways do they deal with them?

We sought to elicit insights from interviews, rather than attempting to reach an objective truth about the ethical impacts of BD + AI. The interpretivist case study approach (Stake 2003) allowed the researchers *‘to understand ‘reality’ as the blending of the various (and sometimes conflicting) perspectives which coexist in social contexts, the common threads that connect the different perspectives and the value systems that give rise to the seeming contradictions and disagreements around the topics discussed. Whether one sees this reality as static (social constructivism) or dynamic (social constructionism) was also a point of consideration, as they both belong in the same “family” approach where methodological flexibility is as important a value as rigour’ (XXX).*

Through extensive brainstorming within the research team, and evaluations of relevant literature, 16 social application domains were established as topics for case study analysis.^7^ The project focused on ten out of these application domains in accordance with the partners’ competencies. The case studies have covered ten domains, and each had their own unique focus, specifications, and niches, which added to the richness of the evaluations (Table 1).

**Table 1:** Case study application domains

No	Case Study Domain	Case Study Focus
CS1	Employee Monitoring and Administration (Antoniou & Andreou, 2019)	A company using the Internet of Things (IoT) for employee monitoring and administration
CS2	Government (Ryan, 2019a)	A division within government, a municipality, using BD + AI
CS3	Agriculture (Ryan, 2019b)	Large agribusiness using BD + AI
CS4	Sustainable Development (Ryan & Gregory, 2019)	1. Large Municipality; 2. Public Organisation; 3. Telecommunications Company; 4. Large Municipality
CS5	Science (Jiya, 2019b)	A large scientific research project
CS6	Insurance (Kancevičienė, 2019)	Health insurance companies
CS7	Energy and Utilities(Hatzakis, Rodrigues, & Wright, 2019)	Energy and utilities company
CS8	Communications, Media, and Entertainment (Macnish, Inguanzo, & Kirichenko, 2019)	Cybersecurity department within a multinational telecommunications company
CS9	Retail and Wholesale Trade (Macnish & Inguanzo, 2019)	A national telecommunications company developing BD + AI for retail customer-relation management
CS10	Manufacturing and natural resources (Jiya, 2019a)	A company developing BD + AI for risk prediction in supply-chain management

The qualitative analysis approach adopted in this study focused on these ten standalone operational case studies that were directly related to the application domains presented in Table 1. These individual case studies provide valuable insights (Yin, 2014, 2015); however, a multiple-case study approach offers a more comprehensive analysis of ethical issues related to BD + AI use (Herriott & Firestone, 1983). Thus, this paper adopts a multiple-case study methodology to identify what insights can be obtained from the ten cases, identifies whether any generalisable understandings can be retrieved, and evaluates how different organisations deal with issues pertaining to BD + AI development and use. The paper does not attempt to derive universal findings from this analysis, in line with the principles of interpretive research, but further attempts to gain an in-depth understanding of the implications of selected BD + AI applications.

The data collection was guided by specific research questions identified through each case, including five desk research questions (see appendix 1); 24 interview questions (see appendix 2); and a checklist of 17 potential ethical issues, developed by the project leader^8^ (see appendix 3). A thematic analysis framework was used to *‘highlight, expose, explore, and record patterns within the collected data. The themes were patterns across data sets that were important to describe several ethical issues which arise through the use of BD* + *AI across different types of organisations and application domains’* (XXX).

**Fig. 2:**
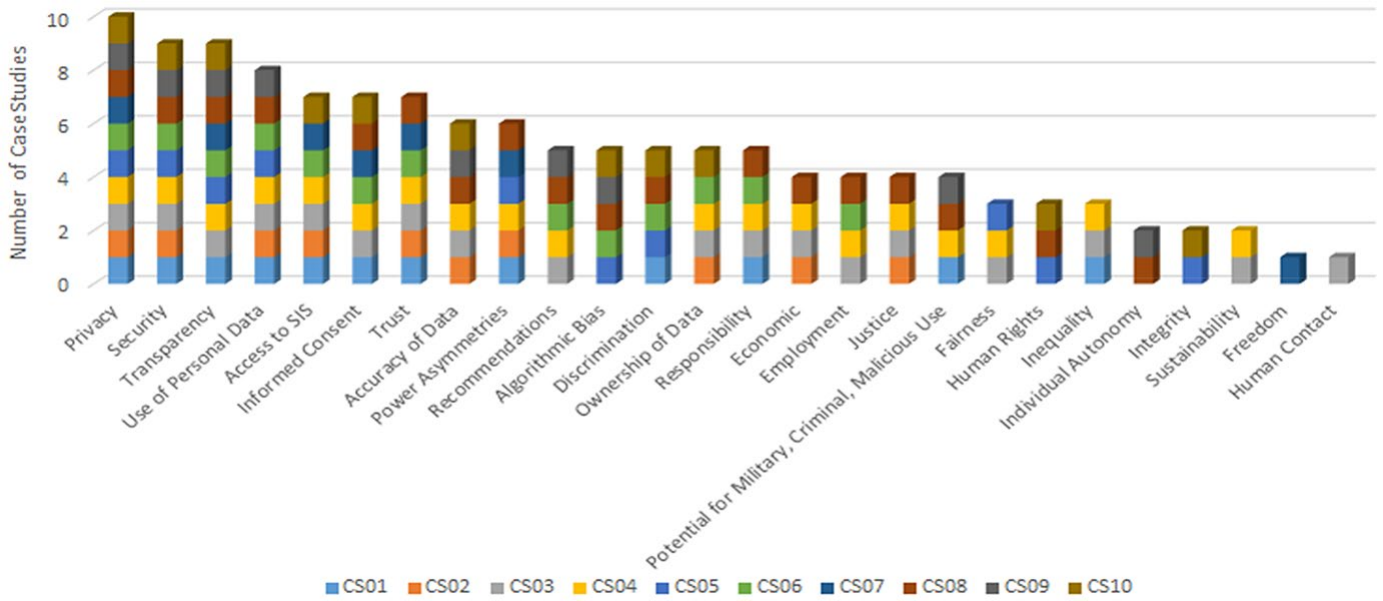
The Prevalence of Ethical Issues in the Case Studies

A workshop was then held after the interviews were carried out. The workshop brought together the experts in the case study team to discuss their findings. This culminated in 26 ethical issues^9^ that were inductively derived from the data collected throughout the interviews (see Fig. 2 and Table 3).^10^ In order to ensure consistency and rigour in the multiple-case study approach, researchers followed a standardised case study protocol (Yin, 2014).^11^

Thirteen different organisations were interviewed for 10 case studies, consisting of 22 interviews in total.^12^ These ranged from 30 min to 1 ½ hours in-person or Skype interviews. The participants that were selected for interviews represented a very broad range of application domains and organisations that use BD + AI. The case study organisations were selected according to their relevance to the overall case study domains and considering their fit with the domains and likelihood of providing interesting insights. The interviewees were then selected according to their ability to explain their BD + AI and its role in their organisation. In addition to interviews, a document review provided supporting information about the organisation. Thus, websites and published material were used to provide background to the research.

## Findings: Ten Case Studies

This section gives a brief overview of the cases, before analysing their similarities and differences. It also highlights the different types of BD + AI being used, and the types of data used by the BD + AI in the case study organisations, before conducting an ethical analysis of the cases. Table 2 presents an overview of the 10 cases to show the roles of the interviewees, the focus of the technologies being used, and the data retrieved by each organisation’s BD + AI. All interviews were conducted in English.

**Table 2:** Case Composition

No	Interviewee(s) Role	Technology Focus	Data Retrieved	Country
CS1	Two members from the Software & Interaction Design Team	Data retrieval, Data interpretation, IoT	Only informative data, no sensitive data	Cyprus
CS2	Project Owner	Data retrieval, machine learning, visualisation dashboards	Public and private transport, phone data, hotel data, tourism and recreation data, and social media data	The Netherlands
CS3	1. Governmental Affairs Management 2. Head of Agronomy Digital Farming 3. Global Sustainability Assessment	Data retrieval, machine learning, visualisation dashboards	Farmers’ data including name, location, contact data Farms’ data including planting, seeding, harvesting times, image data	Germany
CS4	1. CTO Innovation Department 2. Solutions Lab 3. Head of Innovation 4. Chief Digital Officer	1. AI Neuro-Linguistic Programming (NLP) 2. data retrieval/sales 3. data retrieval/use 4. AI text recognition	1. Voluntary: name, number 2. No sensitive data 3. No sensitive data 4. Compulsory: name, car reg, contact info	The Netherlands; Denmark; Germany; and Finland
CS5	1. Biotechnologist 2. Data Scientist 3. Ethicist	AI, super-computing, robotics	Brain science and medicine data	United Kingdom
CS6	1. Lawyer 2. Compliance Officer	Data retrieval, predictive modelling	Organisation Y: access to all national healthcare insurance company’s payment data	Germany
CS7	Two Industry Experts	No AI and limited Big Data usage, focus on smart meters for data retrieval	Energy consumption data, power quality voltage/energy data, and event data	The Netherlands
CS8	Four Senior Security Researchers	Rule-based machine learning data analytics	Personal data from customers and metadata from phone networks	Finland
CS9	CRM AI Lead	Rule-based machine learning data analytics	Their BD + AI handles customer data to improve systems and services	Finland
CS10	1. CEO 2. Marketing Officer	Data retrieval, predictive analytics	Social media data to detect strikes, protests, or political upheaval leading to SCM delays	Austria

The types of organisations that were used in the case studies varied extensively. They included start-ups (CS10), niche software companies (CS1), national health insurers (Organisation X in CS6), national energy providers (CS7), chemical/agricultural multinational (CS3), and national (CS9) and international (CS8) telecommunications providers. The case studies also included public (CS2, Organisation 1 and 4 in CS4) and semi-public (Organisation 2 in CS4) organisations, as well as a large scientific research project (CS5).

The types of individuals interviewed also varied extensively. For example, CS6 and CS7 did not have anyone with a specific technical background, which limited the possibility of analysing issues related to the technology itself. Some case studies *only* had technology experts (such as CS1, CS8, and CS9), who mostly concentrated on technical issues, with much less of a focus on ethical concerns. Other case studies had a combination of both technical and policy-focused experts (i.e. CS3, CS4, and CS5).^13^

Therefore, it must be made fundamentally clear that we are not proposing that all of the interviewees were authorities in the field, or that even collectively they represent a unified authority on the matter, but instead, that we are hoping to show what are the insights and perceived ethical issues of those currently working with AI on the ground view as ethical concerns. While the paper is presenting the ethical concerns found within an array of domains, we do not claim that any individual case study is representative of their entire industry, but instead, our intent was to capture a wide diversity of viewpoints, domains, and applications of AI, to encompass a broad amalgamation of concerns. We should also state that this is not a shortcoming of the study but that it is the normal approach that social science often takes.

The diversity of organisations and their application focus areas also varied. Some organisations focused more so on the Big Data component of their AI, while others more strictly on the AI programming and analytics. Even when organisations concentrated on a specific type of BD + AI, such as Big Data, its use varied immensely, including retrieval (CS1), analysis (CS2), predictive analytics (CS10), and transactional value (Organisation 2 in CS4). Some domains adopted BD + AI earlier and more emphatically than others (such as communications, healthcare, and insurance). Also, the size, investment, and type of organisation played a part in the level of BD + AI innovation (for example, the two large multinationals in CS3 and CS8 had well-developed BD + AI).

The maturity level of BD + AI was also determined by how it was integrated, and its importance, within an organisation. For instance, in organisations where BD + AI were fundamental for the success of the business (e.g. CS1 and CS10), they played a much more important role than in companies where there was less of a reliance (e.g. CS7). In some organisations, even when BD + AI was not central to success, the level of development was still quite advanced because of economic investment capabilities (e.g. CS3 and CS8).

These differences provided important questions to ask throughout this multi-case study analysis, such as: Do certain organisations respond to ethical issues relating to BD + AI in a certain way? Does the type of interviewee affect the ethical issues discussed—e.g. case studies without technical experts, those that *only* had technical experts, and those that had both? Does the type of BD + AI used impact the types of ethical issues discussed? What significance does the type of data retrieved have on ethical issues identified by the organisations? These inductive ethical questions provided a template for the qualitative analysis in the following section.

## Ethical Issues in the Case Studies

Based on the interview data, the ethical issues identified in the case studies were grouped into six specific thematic sections to provide a more conducive, concise, and pragmatic methodology. Those six sections are: control of data, reliability of data, justice, economic issues, role of organisations, and individual freedoms. From the 26 ethical issues, privacy was the only ethical issue addressed in all 10 case studies, which was not surprising because it has received a great deal of attention recently because of the GDPR. Also, security, transparency, and algorithmic bias are regularly discussed in the literature, so we expected them to be significant issues across many of the cases. However, there were many issues that received less attention in the literature—such as access to BD + AI, trust, and power asymmetries—which were discussed frequently in the interviews. In contrast to this, there were ethical issues that were heavily discussed in the literature which received far less attention in the interviews, such as employment, autonomy, and criminal or malicious use of BD + AI (Fig. 2).

The ethical analysis was conducted using a combination of literature reviews and interviews carried out with stakeholders. The purpose of the interviews was to ensure that there were no obvious ethical issues faced by stakeholders in their day-to-day activities which had been missed in the academic literature. As such, the starting point was not an overarching normative theory, which might have meant that we looked for issues which fit well with the theory but ignored anything that fell outside of that theory. Instead the combined approach led to the identification of the 26 ethical issues, each labelled based on particular words or phrases used in the literature or by the interviewees. For example, the term "privacy" was used frequently and so became the label for references to and instances of privacy-relevant concerns. In this section we have clustered issues together based on similar problems faced (e.g. accuracy of data and accuracy of algorithms within the category of ‘reliability of data’).

In an attempt to highlight similar ethical issues and improve the overall analysis to better capture similar perspectives, the research team decided to use the method of clustering, a technique often used in data mining to efficiently group similar elements together. Through discussion in the research team, and bearing in mind that the purpose of the clustering process was to form clusters that would enhance understanding of the impact of these ethical issues, we arrived at the following six clusters: the control of data (covering privacy, security, and informed consent); the reliability of data (accuracy of data and accuracy of algorithms); justice (power asymmetries, justice, discrimination, and bias); economic issues (economic concerns, sustainability, and employment); the role of organisations (trust and responsibility); and human freedoms (autonomy, freedom, and human rights). Both the titles and the precise composition of each cluster of issues are the outcome of a reasoned agreement of the research team. However, it should be clear that we could have used different titles and different clustering. The point is not that each cluster forms a distinct group of ethical issues, independent from any other. Rather the ethical issues faced overlap and play into one another, but to present them in a manageable format we have opted to use this bottom-up clustering approach.

### Human Freedoms

An interviewee from CS10 stated that they were concerned about human rights because they were an integral part of the company’s ethics framework. This was beneficial to their business because they were required to incorporate human rights to receive public funding by the Austrian government. The company ensured that they would not grant *‘full exclusivity on generated social unrest event data to any single party, unless the data is used to minimise the risk of suppression of unrest events, or to protect the violation of human rights’* (XXX). The company demonstrates that while BD + AI has been criticised for infringing upon human rights in the literature, they also offer the opportunity to identify and prevent human rights abuses. The company’s moral framework definitively stemmed from regulatory and funding requirements, which lends itself to the benefit of effective ethical top-down approaches, which is a divisive topic in the literature, with diverging views about whether top-down or bottom-up approaches are better options for improved AI ethics.

### Trust & Responsibility

Responsibility was a concern in 5 of the case studies, confirming the importance it is given in the literature (see Sect. 3). Trust appeared in seven of the case studies. The cases focused on concerns found in the literature, such as BD + AI use in policy development, public distrust about automated decision-making and the integrity of corporations utilising datafication methods (van Dijck 2014).

Trust and control over BD + AI were an issue throughout the case studies. The organisation from the predictive intelligence case study (CS10) identified that their use of social media data raised trust issues. They converged with perspectives found in the literature that when people feel disempowered to use or be part of the BD + AI development process, they tend to lose trust in the BD + AI (Accenture, 2016, 2017). In CS6, stakeholders (health insurers) trusted the decisions made by BD + AI when they were engaged and empowered to give feedback on how their data was used. Trust is enhanced when users can refuse the use of their data (CS7), which correlates with the literature. Companies discussed the benefits of establishing trustworthy relationships. For example, in CS9, they have “*been trying really hard to avoid the existence of fake [mobile phone] base stations, because [these raise] an issue with the trust that people put in their networks”* (XXX).

Corporations need to determine the objective of the data analysis (CS3), what data is required for the BD + AI to work (CS2), and accountability for when it does not work as intended or causes undesirable outcomes (CS4). The issue here is whether the organisation takes direct responsibility for these outcomes, or, if informed consent has been given, can responsibility be shared with the granter of consent (CS3). The cases also raised the question of ‘responsible to whom’, the person whose data is being used or the proxy organisation who has provided data (CS6). For example, in the insurance case study, the company stated that they only had a responsibility towards the proxy organisation and not the sources of the data. All these issues are covered extensively in the literature in most application domains.

### Control of Data

Concerns surrounding the control of data for privacy reasons can be put down to a general awareness of privacy issues in the press, reinforced by the recently-introduced GDPR. This was supported in the cases, where interviewees expressed the opinion that the GDPR had raised general awareness of privacy issues (CS1, CS9) or that it had lent weight to arguments concerning the importance of privacy (CS8).

The discussion of privacy ranged from stressing that it was not an issue for some interviewees, because there was no personal information in the data they used (CS4), to its being an issue for others, but one which was being dealt with (CS2 and CS8). One interviewee (CS5) expressed apprehension that privacy concerns conflicted with scientific innovation, introducing hitherto unforeseen costs. This view is not uncommon in scientific and medical innovation, where harms arising from the use of anonymised medical data are often seen as minimal and the potential benefits significant (Manson & O’Neill, 2007). In other cases (CS1), there was a confusion between anonymisation (data which cannot be traced back to the originating source) and pseudonymisation (where data can be traced back, albeit with difficulty) of users’ data. A common response from the cases was that providing informed consent for the use of personal data waived some of the rights to privacy of the user.

Consent may come in the form of a company contract^14^ or an individual agreement.^15^ In the former, the company often has the advantage of legal support prior to entering a contract and so should be fully aware of the information provided. In individual agreements, though, the individual is less likely to be legally supported, and so may be at risk of exploitation through not reading the information sufficiently (CS3), or of responding without adequate understanding (CS9). In one case (CS5), referring to anonymised data, consent was implied rather than given: the interviewee suggested that those involved in the project may have contributed data without giving clear informed consent. The interviewee also noted that some data may have been shared without the permission, or indeed knowledge, of those contributing individuals. This was acknowledged by the interviewee as a potential issue.

In one case (CS6), data was used without informed consent for fraud detection purposes. The interviewees noted that their organisation was working within the parameters of national and EU legislation, which allows for non-consensual use of data for these ends. One interviewee in this case stated that informed consent was sought for every novel use of the data they held. However, this was sought from the perceived owner of the data (an insurance company) rather than from the originating individuals. This case demonstrates how people may expect their data to be used without having a full understanding of the legal framework under which the data are collected. For example, data relating to individuals may legally be accessed for fraud detection without notifying the individual and without relying on the individual’s consent.

This use of personal data for fraud detection in CS6 also led to concerns regarding opacity. In both CS6 and CS10 there was transparency within the organisations (a shared understanding among staff as to the various uses of the data) but that did not extend to the public outside those organisations. In some cases (CS5) the internal transparency/external opacity meant that those responsible for developing BD + AI were often hard to meet. Of those who were interviewed in CS5, many did not know the providence of the data or the algorithms they were using. Equally, some organisations saw external opacity as integral to the business environment in which they were operating (CS9, CS10) for reasons of commercial advantage. The interviewee in CS9 cautioned that this approach, coupled with a lack of public education and the speed of transformation within the industry, would challenge any meaningful level of public accountability. This would render processes effectively opaque to the public, despite their being transparent to experts.

### Reliability of Data

There can be multiple sources of unreliability in BD + AI. Unreliability originating from faults in the technology can lead to algorithmic bias, which can cause ethical issues such as unfairness, discrimination, and general negative social impact (CS3 and CS6). Considering algorithmic bias as a key input to data reliability, there exist two types of issues that may need to be addressed. Primarily, bias may stem from the input data, referred to as training data, if such data excludes adequate representation of the world, e.g. gender-biased datasets (CS6). Secondly, an inadequate representation of the world may be the result of lack of data, e.g. a correctly designed algorithm to learn from and predict a rare disease, may not have sufficient representative data to achieve correct predictions (CS5). In either case the input data are biased and may result in inaccurate decision-making and recommendations.

The issues of reliability of data stemming from data accuracy and/or algorithmic bias, may escalate depending on their use, as for example in predictive or risk-assessment algorithms (CS10). Consider the risks of unreliable data in employee monitoring situations (CS1), detecting pests and diseases in agriculture (CS3), in human brain research (CS5) or cybersecurity applications (CS8). Such issues are not singular in nature but closely linked to other ethical issues such as information asymmetries, trust, and discrimination. Consequently, the umbrella issue of reliability of data must be approached from different perspectives to ensure the validity of the decision-making processes of the BD + AI.

### Justice

Data may over-represent some people or social groups who are likely to be already privileged or under-represent disadvantaged and vulnerable groups (CS3). Furthermore, people who are better positioned to gain access to data and have the expertise to interpret them may have an unfair advantage over people devoid of such competencies. In addition, BD + AI can work as a tool of disciplinary power, used to evaluate people’s conformity to norms representing the standards of disciplinary systems (CS5). We focus on the following aspects of justice in our case study analysis: power asymmetries, discrimination, inequality, and access.

The fact that issues of power can arise in public as well as private organisations was discussed in our case studies. The smart city case (CS4) showed that the public organisations were aware of potential problems arising from companies using public data and were trying to put legal safeguards in place to avoid such misuse. As a result of misuse, there is the potential that cities, or the companies with which they contract, may use data in harmful or discriminatory ways. Our case study on the use of BD + AI in scientific research showed that the interviewees were acutely aware of the potential of discrimination (CS10). They stated that biases in the data may not be easy to identify, and may lead to misclassification or misinterpretation of findings, which may in turn skew results. Discrimination refers to the recognition of difference, but it may also refer to unjust treatment of different categories of people based on their gender, sex, religion, race, class, or disability. BD + AI are often employed to distinguish between different cases, e.g. between normal and abnormal behaviour in cybersecurity. Determining whether such classification entails discrimination in the latter sense can be difficult, due to the nature of the data and algorithms involved.

Examples of potential inequality based on BD + AI could be seen in several case studies. The agricultural case (CS3) highlighted the power differential between farmers and companies with potential implications for inequality, but also the global inequality between farmers, linked to farming practices in different countries (CS3). Subsistence farmers in developing countries, for example, might find it more difficult to benefit from these technologies than large agro-businesses. The diverging levels of access to BD + AI entail different levels of ability to benefit from them and counteract possible disadvantages (CS3). Some companies restrict access to their data entirely, and others sell access at a fee, while others offer small datasets to university-based researchers (Boyd & Crawford, 2012, p. 674).

### Economic Issues

One economic impact of BD + AI outlined in the agriculture case study (CS3) focused on whether this technology, and their ethical implementation, were economically affordable. If BD + AI could not improve economic efficiency, they would be rejected by the end-user, whether they were more productive, sustainable, and ethical options. This is striking, as it raises a serious challenge for the AI ethics literature and industry. It establishes that no matter how well intentioned and principled AI ethics guidelines and charters are, unless their implementation can be done in an economically viable way, their implementation will be challenged and resisted by those footing the bill.

The telecommunications case study (CS9) focused on how GDPR legislation may economically impact businesses using BD + AI by creating disparities in competitiveness between EU and non-EU companies developing BD + AI. Owing to the larger data pools of the latter, their BD + AI may prove to be more effective than European-manufactured alternatives, which cannot bypass the ethical boundaries of European law in the same way (CS8). This is something that is also being addressed in the literature and is a very serious concern for the future profitability and development of AI in Europe (Wallace & Castro, 2018). The literature notes additional issues in this area that were not covered in the cases. There is the potential that the GDPR will increase costs of European AI companies by having to manually review algorithmic decision-making; the right to explanation could reduce AI accuracy; and the right to erasure could damage AI systems (Wallace & Castro, 2018, p. 2).

One interviewee stated that public–private BD + AI projects should be conducted in a collaborative manner, rather than a sale-of-service (CS4). However, this harmonious partnership is often not possible. Another interviewee discussed the tension between public and private interests on their project—while the municipality tried to focus on citizen value, the ICT company focused on the project’s economic success. The interviewee stated that the project would have terminated earlier if it were the company’s decision, because it was unprofitable (CS4). This is a huge concern in the literature, whereby private interests will cloud, influence, and damage public decision-making within the city because of their sometimes-incompatible goals (citizen value vs. economic growth) (Sadowski & Pasquale, 2015). One interviewee said that the municipality officials were aware of the problems of corporate influence and thus are attempting to implement the approach of ‘data sovereignty’ (CS2).

During our interviews, some viewed BD + AI as complementary to human employment (CS3), collaborative with such employment (CS4), or as a replacement to employment (CS6). The interviewees from the agriculture case study (CS3) stated that their BD + AI were not sufficiently advanced to replace humans and were meant to complement the agronomist, rather than replace them. However, they did not indicate what would happen when the technology *is* advanced enough, and it becomes profitable to replace the agronomist. The insurance company interviewee (CS6) stated that they use BD + AI to reduce flaws in personal judgment. The literature also supports this viewpoint, where BD + AI is seen to offer the potential to evaluate cases impartially, which is beneficial to the insurance industry (Belliveau, Gray, & Wilson, 2019).^16^ The interviewee reiterated this and also stated that BD + AI would reduce the number of people required to work on fraud cases. The interviewee stated that BD + AI are designed to replace these individuals, but did not indicate whether their jobs were secure or whether they would be retrained for different positions, highlighting a concern found in the literature about the replacement and unemployment of workers by AI (Bossman, 2016). In contrast to this, a municipality interviewee from CS4 stated that their chat-bots are used in a *collaborative* way to assist customer service agents, allowing them to concentrate on higher-level tasks, and that there are clear policies set in place to protect their jobs.

Sustainability was only explicitly discussed in two interviews (CS3 and CS4). The agriculture interviewees stated that they wanted to be the ‘first’ to incorporate sustainability metrics into agricultural BD + AI, indicating a competitive and innovative rationale for their company (CS3). Whereas the interviewee from the sustainable development case study (CS4) stated that their goal of using BD + AI was to reduce Co2 emissions and improve energy and air quality. He stated that there are often tensions between ecological and economic goals and that this tension tends to slow down the efforts of BD + AI public–private projects—an observation also supported by the literature (Keeso, 2014). This tension between public and private interests in BD + AI projects was a recurring issue throughout the cases, which will be the focus of the next section on the role of organisations.

## Discussion and Conclusion

The motivation behind this paper is to come to a better understanding of ethical issues related to BD + AI based on a rich empirical basis across different application domains. The exploratory and interpretive approach chosen for this study means that we cannot generalise from our research to all possible examples of BD + AI, but it does allow us to generalise to theory and rich insights (Walsham, 1995a, b, 2006). These theoretical insights can then provide the basis for further empirical research, possibly using other methods to allow an even wider set of inputs to move beyond some of the limitations of the current study.

### Organisational Practice and the Literature

The first point worth stating is that there is a high level of consistency both among the case studies and between cases and literature. Many of the ethical issues identified cut across the cases and are interpreted in similar ways by different stakeholders. The frequency distribution of ethical issues indicates that very few, if any, issues are relevant to all cases but many, such as privacy, have a high level of prevalence. Despite appearing in all case studies, privacy was not seen as overly problematic and could be dealt with in the context of current regulatory principles (GDPR). Most of the issues that we found in the literature (see Sect. 2) were also present in the case studies. In addition to privacy and data protection, this included accuracy, reliability, economic and power imbalances, justice, employment, discrimination and bias, autonomy and human rights and freedoms.

Beyond the general confirmation of the relevance of topics discussed in the literature, though, the case studies provide some further interesting insights. From the perspective of an individual case some societal factors are taken for granted and outside of the control of individual actors. For example, intellectual property regimes have significant and well-recognised consequences for justice, as demonstrated in the literature. However, there is often little that individuals or organisations can do about them. Even in cases where individuals may be able to make a difference and the problem is clear, it is not always obvious how to do this. Some well-publicised discrimination cases may be easy to recognise, for example where an HR system discriminates against women or where a facial recognition system discriminates against black people. But in many cases, it may be exceedingly difficult to recognise discrimination where it is not clear how a person is discriminated against. If, for example, an image-based medical diagnostic system leads to disadvantages for people with genetic profiles, this may not be easy to identify.

With regards to the classification of the literature suggested in Sect. 2 along the temporal dimension, we can see that the attention of the case study respondents seems to be correlated to the temporal horizon of the issues. The issues we see as short-term figures most prominently, whereas the medium-term issues, while still relevant and recognisable, appear to be less pronounced. The long-term questions are least visible in the cases. This is not very surprising, as the short-term issues are those that are at least potentially capable of being addressed relatively quickly and thus must be accessible on the local level. Organisations deploying or using AI therefore are likely to have a responsibility to address these issues and our case studies have shown that they are aware of this and putting measures in place. This is clearly true for data protection or security issues. The medium-term issues that are less likely to find local resolutions still figure prominently, even though an individual organisation has less influence on how they can be addressed. Examples of this would be questions of unemployment, justice, or fairness. There was little reference to what we call long-term issues, which can partly be explained by the fact that the type of AI user organisations we investigated have very limited influence on how they are perceived and how they may be addressed.

### Interpretative Differences on Ethical Issues

Despite general agreement on the terminology used to describe ethical issues, there are often important differences in interpretation and understanding. In the first ethics theme, control of data, the perceptions of privacy ranged from ‘not an issue’ to an issue that was being dealt with. Some of this arose from the question of informed consent and the GDPR. However, a reliance on legislation, such as GDPR, without full knowledge of the intricacies of its details (i.e. that informed consent is only one of several legal bases of lawful data processing), may give rise to a false sense of security over people’s perceived privacy. This was also linked to the issue of transparency (of processes dealing with data), which may be external to the organisation (do people outside understand how an organisation holds and processes their data), or internal (how well does the organisation understand the algorithms developed internally) and sometimes involve deliberate opacity (used in specific contexts where it is perceived as necessary, such as in monitoring political unrest and its possible consequences). Therefore, a clearer and more nuanced understanding of privacy and other ethical terms raised here might well be useful, albeit tricky to derive in a public setting (for an example of complications in defining privacy, see Macnish, 2018).

Some issues from the literature were not mentioned in the cases, such as warfare. This can easily be explained by our choice of case studies, none of which drew on work done in this area. It indicates that even a set of 10 case studies falls short of covering all issues.

A further empirical insight is in the category we called ‘role of organisations’, which covers trust and responsibility. Trust is a key term in the discussion of the ethics of AI, prominently highlighted by the focus on trustworthy AI by the EU’s High-Level Expert Group, among others. We put this into the ‘role of organisations’ category because our interaction with the case study respondents suggested that they felt it was part of the role of their organisations to foster trust and establish responsibilities. But we are open to the suggestion that these are concepts on a slightly different level that may provide the link between specific issues in applications and broader societal debate.

### Next Steps: Addressing the Ethics of AI and Big Data

This paper is predominantly descriptive, and it aims to provide a theoretically sound and empirically rich account of ethical concerns in AI + BD. While we hope that it proves to be insightful it is only a first step in the broader journey towards addressing and resolving these issues. The categorisation suggested here gives an initial indication of which type of actor may be called upon to address which type of issue. The distinction between micro-, meso- and macro perspectives suggested by Haenlein and Kaplan (2019) resonates to some degree with our categorisation of issues.

This points to the question what can be done to address these ethical issues and by whom should it be done? We have not touched on this question in the theoretical or empirical part of the paper, but the question of mitigation is the motivating force behind much of the AI + BD ethics research. The purpose of understanding these ethical questions is to find ways of addressing them.

This calls for a more detailed investigation of the ethical nature of the issues described here. As indicated earlier, we did not begin with a specific ethical theoretical framework imposed onto the case studies, but did have some derived ethics concepts which we explored within the context of the cases and allowed others to emerge over the course of the interviews. One issue is the philosophical question whether the different ethical issues discussed here are of a similar or comparable nature and what characterises them as ethical issues. This is not only a philosophical question but also a practical one for policymakers and decision makers. We have alluded to the idea that privacy and data protection are ethical issues, but they also have strong legal implications and can also be human rights issues. It would therefore be beneficial to undertake a further analysis to investigate which of these ethical issues are already regulated and to what degree current regulation covers BD + AI, and how this varies across the various EU nations and beyond.

Another step could be to expand an investigation like the one presented here to cover the ethics of AI + BD debate with a focus on suggested resolutions and policies. This could be achieved by adopting the categorisation and structure presented here and extending it to the currently discussed option for addressing the ethical issues. These include individual and collective activities ranging from technical measures to measure bias in data or individual professional guidance to standardisation, legislation, the creation of a specific regulator and many more. It will be important to understand how these measures are conceptualised as well as which ones are already used to which effect. Any such future work, however, will need to be based on a sound understanding of the issues themselves, which this paper contributes to. The key contribution of the paper, namely the presentation of empirical findings from 10 case studies show in more detail how ethical issues play out in practice. While this work can and should be expanded by including an even broader variety of cases and could be supplemented by other empirical research methods, it marks an important step in the development of our understanding of these ethical issues. This should form a part of the broader societal debate about what these new technologies can and should be used for and how we can ensure that their consequences are beneficial for individuals and society.

## Appendices

### Appendix 1: Desk Research Questions

Number Research Question.

1. In which sector is the organisation located (e.g. industry, government, NGO, etc.)?
2. What is the name of the organisation?
3. What is the geographic scope of the organisation?
4. What is the name of the interviewee?
5. What is the interviewee’s role within the organisation?

### Appendix 2: Interview Research Questions

No Research Question.

1. What involvement has the interviewee had with BD + AI within the organisation?
2. What type of BD + AI is the organisation using? (e.g. IBM Watson, Google Deepmind)
3. What is the field of application of the BD + AI (e.g. administration, healthcare, retail)
4. Does the BD + AI work as intended or are there problems with its operation?
5. What are the innovative elements introduced by the BD + AI (e.g. what has the technology enabled within the organisation?)
6. What is the level of maturity of the BD + AI ? (i.e. has the technology been used for long at the organisation? Is it a recent development or an established approach?)
7. How does the BD + AI interact with other technologies within the organisation?
8. What are the parameters/inputs used to inform the BD + AI ? (e.g. which sorts of data are input, how is the data understood within the algorithm?). Does the BD + AI collect and/or use data which identifies or can be used to identify a living person (personal data)?. Does the BD + AI collect personal data without the consent of the person to whom those data relate?
9. What are the principles informing the algorithm used in the BD + AI (e.g. does the algorithm assume that people walk in similar ways, does it assume that loitering involves not moving outside a particular radius in a particular time frame?). Does the BD + AI classify people into groups? If so, how are these groups determined? Does the BD + AI identify abnormal behaviour? If so, what is abnormal behaviour to the BD + AI ?
10. Are there policies in place governing the use of the BD + AI ?
11. How transparent is the technology to administrators within the organisation, to users within the organisation?
12. Who are the stakeholders in the organisation?
13. What has been the impact of the BD + AI on stakeholders?
14. How transparent is the technology to people outside the organisation?
15. Are those stakeholders engaged with the BD + AI ? (e.g. are those affected aware of the BD + AI, do they have any say in its operation?). If so, what is the nature of this engagement? (focus groups, feedback, etc.)
16. In what way are stakeholders impacted by the BD + AI ? (e.g. what is the societal impact: are there issues of inequality, fairness, safety, filter bubbles, etc.?)
17. What are the costs of using the BD + AI to stakeholders? (e.g. potential loss of privacy, loss of potential to sell information, potential loss of reputation)
18. What is the expected longevity of this impact? (e.g. is this expected to be temporary or long-term?)
19. What has been the impact of the BD + AI on stakeholders?
20. Are those stakeholders engaged with the BD + AI ? (e.g. are those affected aware of the BD + AI, do they have any say in its operation?)
21. If so, what is the nature of this engagement? (focus groups, feedback, etc.)
22. In what way are stakeholders impacted by the BD + AI ? (e.g. what is the societal impact: are there issues of inequality, fairness, safety, filter bubbles, etc.?)
23. What are the costs of using the BD + AI to stakeholders? (e.g. potential loss of privacy, loss of potential to sell information, potential loss of reputation)
24. What is the expected longevity of this impact? (e.g. is this expected to be temporary or long-term?)

### Appendix 3: Checklist of Ethical Issues

**Table:**

Ethical Issue	Question Example	√
Privacy	Does the use of the technology raise concerns that people’s privacy might be at risk or endangered?	
Personal Data	Does the technology or its use presume a particular group or person “own” the data? If so, who?	
Security	Does the technology use personally-identifying data? If so, is this data stored and treated securely?	
Inclusion of stakeholders	Are people affected by the technology involved in any way with its use or implementation? Do they have an opportunity to have a say in how the technology impacts them?	
Consent of stakeholders	Have people affected by the technology been given an opportunity to consent to that technology existing or having the impact that it does on their lives?	
Loss of employment	Does the use of the technology put people’s jobs at risk, either directly or indirectly?	
Autonomy/agency	Does the use of the technology impact in any way on people’s freedom to choose how to live their lives?	
Discrimination	Can/does the technology or its use lead to discriminating behaviour in any way? Does the technology draw on data sets that are representative of those stakeholders affected by the technology?	
Potential for military/criminal/nefarious use	Could the technology be used for military, criminal or other ends which were not envisaged or intended by its developers?	
Trust	Does the technology impact people’s trust in organisations, other people, or the technology itself?	
Power asymmetries	Can or does the technology exacerbate existing power asymmetries by, for instance, giving a large amount of power to those already holding power over other people?	
Inequality	Can or does the technology reduce inequalities in society or exacerbate them?	
Fairness	Is the technology fair in the way in which it treats those affected by it? Are there unfair practices which arise in relation to the technology?	
Justice	Does the technology or its use raise a feeling of injustice on the part of one or more groups affected?	
Freedom	Does the technology or its use raise questions regarding freedom of speech, censorship, or freedom of assembly?	
Sustainability	Is the technology or its use sustainable, or does it draw on limited natural resources in some way?	
Environmental impact	Does the technology have any impact on the environment, and if so what?	

## Footnotes

1 Throughout the paper, XXX will be used to anonymise relevant text that may identify the authors, either through the project and/or publications resulting from the individual case studies. All case studies have been published individually. Several the XXX references in the findings refer to these individual publications which provide more detail on the cases than can be provided in this cross-case analysis.

2 The ethical issues that we discussed throughout the case studies refers to issues broadly construed as ethical issues, or issues that have ethical significance. While some issues may not be directly obvious how they are ethical issues, they may give rise to significant harm relevant to ethics. For example, accuracy of data may not explicitly be an ethical issue, if inaccurate data is used in algorithms, it may lead to discrimination, unfair bias, or harms to individuals.

3 Such as chat-bots, natural language processing AI, IoT data retrieval, predictive risk analysis, cybersecurity machine-learning, and large dataset exchanges.

4 https://ec.europa.eu/futurium/en/ai-alliance-consultation/guidelines/1.

5 https://ec.europa.eu/digital-single-market/en/high-level-expert-group-artificial-intelligence.

6 The type of AI currently in vogue, as outlined earlier, is based on machine learning, typically employing artificial neural networks for big data analysis. This is typically seen as ‘narrow AI’ and it is not clear whether there is a way from narrow to general AI, even if one were to accept that achieving general AI is fundamentally possible.

7 The 16 social domains were: Banking and securities; Healthcare; Insurance; Retail and wholesale trade; Science; Education; Energy and utilities; Manufacturing and natural resources; Agriculture; Communications, media and entertainment; Transportation; Employee monitoring and administration; Government; Law enforcement and justice; Sustainable development; and Defence and national security.

8 This increased to 26 ethical issues following a group brainstorming session at the case study workshop.

9 The nine additional ethical issues from the initial 17 drafted by the project leader were: human rights, transparency, responsibility, ownership of data, algorithmic bias, integrity, human rights, human contact, and accuracy of data.

10 The additional ethical issues were access to BD + AI, accuracy of data, accuracy of recommendations, algorithmic bias, economic, human contact, human rights, integrity, ownership of data, responsibility, and transparency. Two of the initial ethical concerns were removed (inclusion of stakeholders and environmental impact). The issues raised concerning inclusion of stakeholders were deemed to be sufficiently included in access to BD + AI, and those relating to environmental impact were felt to be sufficiently covered by sustainability.

11 The three appendices attached in this paper comprise much of this case study protocol.

12 CS4 evaluated four organisations, but one of these organisations was also part of CS2 – Organisation 1. CS6 analysed two insurance organisations.

13 Starting out, we aimed to have both policy/ethics-focused experts within the organisation and individuals that could also speak with us about the technical aspects of the organisation’s BD + AI. However, this was often not possible, due to availability, organisations’ inability to free up resources (e.g. employee’s time) for interviews, or lack of designated experts in those areas.

14 For example, in CS1, CS6, and CS8.

15 For example, in CS2, CS3, CS4, CS5, CS6, and CS9.

16 As is discussed elsewhere in this paper, algorithms also hold the possibility of reinforcing our prejudices and biases or creating new ones entirely.
